# Sacroiliac Joint Fusion: A Comparison Between Robotic Guidance Fusion and Minimally Invasive Navigation Fusion

**DOI:** 10.7759/cureus.90744

**Published:** 2025-08-22

**Authors:** Abigail Peterson, Ataollah Shahbandi, Hailey Mattheisen, Pegah Ghamasaee, Abdul Mounnem Yassin Kassab, Mohamad Bakhaidar, Saman Shabani

**Affiliations:** 1 Neurosurgery, Medical College of Wisconsin, Milwaukee, USA; 2 Surgery, King Abdulaziz University Faculty of Medicine, Jeddah, SAU

**Keywords:** arthrodesis, minimally invasive surgical procedures, neuronavigation, robotic surgical procedures, sacroiliac joint

## Abstract

Background and objective

Sacroiliac joint (SIJ) dysfunction, a frequently underdiagnosed cause of low back pain, remains relatively understudied. Minimally invasive methods, including navigation and robotic technology, are gaining popularity due to their lower surgical risks. This study aimed to compare outcomes of navigation-assisted (NA) SIJ fusion and robotic-assisted (RA) SIJ fusion performed at our institution.

Methods

We retrospectively reviewed adult patients who underwent SIJ fusion between November 2022 and September 2024. Details of demographic data, operative details (OR time, screw placement accuracy), postoperative complications, and pain scores (preoperative, postoperative) were collected. Changes in pain scores and surgical metrics were compared between the NA and RA groups.

Results

Our study included 31 patients who underwent SIJ fusion during the study period: 19 with NA and 12 with RA. Both cohorts were comparable at baseline in age (p=0.416), BMI (p=0.478), and prior surgeries (p=0.879). The group comprised 21 females and 10 males, with an average age of 65.5 years. Operative time was significantly longer in the RA group (p<0.001). Regarding screw placement accuracy, no intraoperative screw misplacements occurred in either group. The NA cohort had 10.3% improvement in their pain scores within 30 days postoperatively, while the RA group had 31.0% improvement (p=0.52). No postoperative complication necessitating a revision surgery occurred in the patient cohort. Two hardware failures (screw fractures) were observed in the NA group without worsening of symptoms. Long-term pain outcomes were also comparable, showing an improvement of around 5% in the RA group and 62% in the NA group (p=0.083).

Conclusions

While there was no statistically significant difference between the groups, a trend toward lower postoperative pain scores was observed in the RA group. The extended operative time in the RA group is attributable to the new technique at our hospital.

## Introduction

The sacroiliac joint (SIJ) is a large joint that primarily provides biomechanical stability, supported by the surrounding sacroiliac ligaments, while permitting only minimal motion in various directions [1]. Low back pain is a highly prevalent condition - up to 30% in the general population - leading to substantial economic and healthcare impacts with billions spent annually for its treatment [2,3]. SIJ dysfunction has been a subject of interest in the study of back pain, as it contributes to an estimated 10-38% of cases of low back pain [2], affecting up to 25% of patients with persistent low back pain below the L5 region [4,5]. This condition is strongly associated with older age and is more common in the male sex [6]. The SIJ undergoes varying changes with aging, starting with morphological degradation, such as osteoarthritic degeneration and chondrocyte clustering, leading to restriction of motion beginning in the third decade [3]. Abnormal mobility of the SIJ causes the pelvis to tilt, requiring the abdominal muscles to compensate by stabilizing rotation and absorbing the joint’s force.

SIJ fusion, a surgical intervention to stabilize and fuse the SIJ, has been proposed as a treatment option for refractory SIJ dysfunction [3,7]. SIJ fusion provides pain relief without worsening pain or disability [6, 8-9]. Surgical stabilization of the SIJ can include an open technique or an X-ray-guided approach using screws and cages, minimally invasive surgery (MIS) with navigation (NA) or robotic assistance (RA), and several other methods that aim to stabilize the SIJ and improve patient outcomes [10]. An open SIJ fusion approach has been associated with higher morbidity due to greater tissue exposure, making a minimally invasive approach more extensively adopted. However, this procedure’s failure rate is high, with some studies finding that 30% of patients experience persistent SIJ pain following fusion with any of these approaches [5,6]. Modern approaches with spinal robotic technology have become increasingly popular due to enhanced accuracy with screw placement [11]. While some studies found no differences between NA and RA SIJ fusion screw placement accuracy and postoperative outcomes, the robotic approach may reduce radiation exposure [6,12].

There is a paucity of data comparing minimally invasive, NA SIJ fusion to robotic-assisted RA SIJ fusion. Therefore, this study aims to present our center's experience with NA and RA SIJ fusion. We engage in a discussion of the SIJ fusion surgical techniques, focusing on the use of the Mazor™ robotic guidance system (MedtronicTM, Minneapolis, MN) and comparing patient outcomes with these two approaches. The primary objective of this study was to compare postoperative outcomes following NA versus RA SIJ fusion.

## Materials and methods

Study design

We conducted a retrospective review of our institutional database for all SIJ fusion cases performed between September 2022 and December 2024. Our institution began utilizing the RA technique during the current study period, aligning with the timing of patient recruitment. All eligible adult patients undergoing SIJ fusion for SIJ dysfunction were included. SIJ dysfunction was defined as persistent pain in the SIJ despite multiple conservative interventions. Patients diagnosed with SIJ dysfunction through clinical assessments were referred for SIJ injections and subsequently evaluated to determine their response. Patients demonstrating a positive response to the injections were considered candidates for SIJ fusion. We excluded patients who underwent SIJ fusion in the setting of trauma, tumor, or as part of instrumented spinal fusion. This study was approved by the Institutional Review Board. This study was conducted in accordance with Strengthening the Reporting of Observational studies in Epidemiology (STROBE) guidelines. Data used for this study is available upon reasonable request.

Variables

We collected data on patient demographics, comorbidities, and surgical details. Comorbidities encompassed conditions such as chronic low back pain, obesity, and a previous history of spinal surgery. The primary outcome of this study was pain improvement, and the secondary outcomes were operative time, screw accuracy, and complication rates. Perioperative data such as operative time, pain scores (preoperative, post-anesthesia care unit (PACU), and postoperative), and follow-up periods were collected. Preoperative pain scores were recorded based on a pre-procedure protocol completed one day before surgical intervention; we reported the average pain scores recorded as the measure for preoperative pain, the other options being pain at rest and pain during activity. Pain outcomes were estimated by comparing the preoperative and postoperative pain scores in each cohort, with postoperative pain being recorded in the most recent follow-up visit. Operative time was calculated as the time from the first incision to the time of incision closure. Additionally, when available, we collected follow-up data at 30 days, as well as the most recent follow-up time points (in months) and long-term pain scores, which were characterized either qualitatively as improved, worsened, or stable, or quantitatively using a standardized visual analog scale. Relevant pain scores were reported on a scale from 0 to 10 and were reported both in the PACU and at follow-up visits that occurred within a few months of the procedure.

Statistical analysis

A statistical comparison of baseline characteristics and collected pain scores was performed between the NA and RA groups to evaluate the efficacy of each surgical technique. Data analysis was done using IBM SPSS Statistics version 29 (IBM Corp., Armonk, NY) [13]. The Kolmogorov-Smirnov test was used to determine the normality of the data and the Mann-Whitney U test for univariate analysis of quantitative data, including age, BMI, all pain scores (preoperative, PACU, 30-day postoperative and latest follow-up pain scores, in addition to percent change), number of screws placed, operative time, and follow-up period in months. Qualitative data were compared with Fisher's exact test between NA and RA groups regarding sex, previous spine surgeries, and 30-day complications.

Surgical technique

NA Group

After induction of general anesthesia and prone positioning on the operating table, the area was prepped and draped in a standard sterile fashion. The posterior superior iliac spine (PSIS) was then palpated to locate its superior lateral margin, followed by percutaneous placement of a reference frame, which remained fixed throughout the procedure. It is crucial to ensure that the fixed reference frame remains stationary post-imaging to maintain navigation accuracy, as any displacement could necessitate repeating the imaging process. Imaging data acquisition was performed using an O-arm, ensuring the inclusion of the pelvis and lumbar spine. To minimize radiation exposure, all operating room personnel exited the room during this step. The Stealth Navigation System (Medtronic™, Minneapolis, MN) was used for both imaging data acquisition and processing throughout the surgical procedure.

Using the navigation system, the starting point and trajectory for the screw insertion were established, and skin incision markings were made. The skin and muscle were incised using a blade, and the trajectory was reconfirmed using navigation. Drilling was performed from the ilium to the sacrum, crossing the SI joint. The entry point and trajectory of the drilling course were saved using the Stealth Navigation System. Multiple taps were performed, starting with a smaller tap and progressively increasing to a tap just 1 mm smaller in diameter than the SIJ screw, all under real-time multiplanar navigation. The pathway was then palpated using a pedicle probe to ensure no bone violation along the screws had occurred. Once confirmed, an SIJ screw was then placed using the same technique with navigation guidance. Then a second screw is inserted following the same steps. To verify proper placement, intraoperative anterior-posterior and lateral X-rays were acquired using a C-arm after instrumentation.

RA Group

In the RA group, thin-slice (1 mm) CT scans were used for preoperative planning. The CT scan was uploaded into the Mazor™ robotic system's interactive software to determine the ideal screw size, diameter, and placement trajectories across sagittal, coronal, and axial planes. Alternatively, surgical planning can be carried out intraoperatively using an O-arm, eliminating the need for a preoperative CT scan. The planning software provides a three-dimensional evaluation of each vertebra, using a proprietary algorithm for anatomical landmark recognition to enhance accuracy and predictability.

We positioned the patient prone, and the field was prepped and draped in a sterile fashion, similar to the NA group. The robotic arm was assembled and draped in a sterile fashion, and the robotic system was connected to the operating table via a bed frame adapter. A Schanz pin was then inserted into the PSIS, and the robotic arm was connected to it via a bone mount bridge, facilitating connection to the patient. We then initiated a series of steps to synchronize the robotic arm with the patient’s anatomy. Fluoroscopic images (anterior-posterior and oblique/lateral views) were captured to register the robotic system and properly align the preoperative CT scans with the patient’s anatomy. Once this was completed, the navigation's accuracy was assessed. Following the preplanned trajectory, the robotic arm was sent to the designated entry point. A skin blade, through the robotic arm cannula, was used to incise the skin and muscle down to the bone. A drill was then positioned through the cannula, and the entry point and trajectory were reconfirmed. The drill was typically started off-bone to decrease the risk of skiving and then advanced to create the pilot hole. Once completed, the screw hole was tapped, and a ball-tipped probe was used to check for any bone perforation. Screws were then inserted under direct 3D navigation by the physician. Similarly, a second screw was then inserted following the same steps. Anteroposterior and lateral X-rays were utilized to confirm the location of the screws and compare them to the preoperative plan.

## Results

Participants

We identified 31 patients who underwent SIJ fusion at our institution during the study period, 19 in the NA group and 12 in the RA group. This cohort included 21 (67.7%) females and 10 (32.3 %) males with an average age of 65.5 years. Among the included patients, we found that 19 patients (61.3%) were classified as obese (BMI >30), and 21 (67.7%) were diagnosed with chronic back pain. A total of 19 (61.3%) of patients had undergone prior spinal surgery, including four (12.9%) with decompression procedures, seven (22.6%) with two-level spinal fusion, and eight (25.8%) with fusion involving more than two levels. 

No differences were found in the preoperative pain scores, with the average preoperative pain score being 5.9 in the NA group and 6.2 in the RA group (p=0.86). Preoperatively, opioid use for pain management was reported in 18 (94.7%) patients in the NA group and eight (66.7%) in the RA group (p=0.04), which represented a statistically significant difference.

Operative variables

Laterally, an average of 2.5 screws was placed in the NA group and an average of 2.0 screws in the RA group. Most patients had left or right SIJ fusion; six (31.6%) in the NA group and one (8.3%) in the RA group had bilateral screws placed. Operative times were significantly shorter in the NA group, averaging 64.1 minutes, compared to the RA group, which averaged 103.2 minutes (p=0.003).

Outcomes

Pain outcomes in the NA group decreased by an average of 6.5% and by 29.2% in the RA group (p=0.76). All patients in our study were prescribed opioids upon discharge. Only four patients - two (11%) in the NA group and two (16%) in the RA group - experienced a 30-day complication readmission. Each patient presented for readmission with different complaints: two in the NA group with left buttock pain and difficulty ambulating, and two in the RA group with blood in the stool and drainage from the surgical site. The mean postoperative follow-up duration was 18.50 months for the NA group and 12.04 months for the RA group (p=0.834). In the NA group, 11 of 19 patients (58%) reported improvement in pain, two (10%) experienced worsening, and six (32%) remained stable. Similarly, in the RA group, seven of 12 patients (58%) reported improved pain, while five (41%) remained stable; no patients in this group reported worsening symptoms. There was no statistically significant difference in follow-up pain scores between the groups (p=0.834).

Numerical postoperative pain scores were available for 13 patients. The NA group showed a 5% reduction in pain scores, whereas the RA group demonstrated a 62% reduction at the most recent follow-up (p=0.08). Regarding the navigation accuracy, no screw misplacements were identified intraoperatively; however, two hardware failures - both screw fractures - were observed in the NA group, with no associated worsening of symptoms. There were no complications that required revision procedures in either group. The demographic, preoperative, and postoperative data collected in this study are summarized in Table 1.

**Table 1:** Comparison of patient demographics and clinical outcomes between surgical cohorts ^*^Statistically significant Pain scores were averaged for each patient based on available data. Postoperative pain scores were calculated for patients during their follow-up appointment, the period indicated in the last row of the table BMI: body mass index; F: female; M: male; OR: operating room; PACU: post-anesthesia care unit; SD: standard deviation; IQR: interquartile range

Characteristics	NA group (n=19)	RA group (n=12)	P-value
Age, years, mean (SD)	67.21 (17.50)	64.92 (29.25)	0.72
Sex, n (%)	6 M (31.6%)/13 F (68.4%)	4 M (33.3%)/8 F (66.7%)	0.92
BMI, kg/m^2^, mean (SD)	33.4 (11.44)	35.5 (11.14)	0.69
Previous spine surgery, n (%)	12 (63.2%)	7 (58.3%)	0.79
Preoperative opioid, n (%)	18 (94.7%)	8 (66.7%)	0.04^*^
Pain scores			
Preoperative, mean (SD)	5.9 (2.60)	6.2 (1.82)	0.86
PACU, mean (SD)	4.4 (1.78)	3.9 (2.16)	0.50
Postoperative, mean (SD)	5.3 (2.16)	5.5 (3.62)	0.82
Absolute change in pain score at 30 days, mean (SD)	0.73 (3.52)	1.36 (3.08)	0.70
30-day percentage decrease, median (IQR)	50% (97.50)	85.7% (60.25)	0.76
Absolute change in pain score at latest follow-up, mean (SD)	2.2 (3.56)	4.64 (3.51)	0.27
Latest follow-up percentage decrease, median (IQR)	0% (4.2)	20% (6.5)	0.07
Procedural data			
Number of screws, mean (SD)	2.5 (2)	2 (2)	0.83
Total time in OR, minutes, mean (SD)	64.1 (14.4)	103.2 (51)	<0.01^*^
30-day complications, n (%)	2 (10.5%)	2 (16.7%)	0.21
Postoperative data			
Follow-up period months, mean (SD)	18.50 (6.38)	12.04 (6.04)	0. 53

## Discussion

This study addresses a gap in the medical literature by comparing surgical outcomes of the Mazor™ robotic system with minimally invasive, NA SIJ fusion. In this retrospective analysis of 31 patients, no significant difference in pain score reduction was found between the NA and RA groups 30 days postoperatively (6.5% vs. 29.2%, p=0.76) and in follow-up appointments (5% vs 62%, p=0.07) despite greater improvement in the RA group in both postoperative periods. Significant differences were observed in preoperative opioid use (94.7% in the NA and 67% RA group, p=0.04) and operative time, which was longer in the RA group (p=0.003). Preoperative opioid use differed between the cohorts, which may impact the interpretation of the pain outcomes.

The RA group showed a statistically significant longer OR time, which can primarily be attributed to the initial integration of robotic technology in our hospital and the consequent increase in setup duration. The longer OR time has been reported in other studies investigating robotic SIJ fusion [8,12]. As technological advancements in robotics continue and we continue to build our operational experience, it is expected that setup times will get shorter. Consistent with this, other studies have shown a trend toward shorter times with increased experience in robotic-assisted spine surgery [14].

Although postoperative pain improvement did not differ significantly between groups, the interpretation of this data is limited by confounding variables, most notably the significantly higher rate of preoperative opioid use in the NA cohort. Chronic opioid use is associated with reduced postoperative analgesic efficacy, opioid-induced hyperalgesia, and poorer functional outcomes [15,16]. One retrospective study analyzing SIJ fusion outcomes found patients with a history of chronic opioid use were 8.5 times less likely to achieve meaningful improvement in pain and disability following surgery [17]. This imbalance in our cohort likely contributed to our nonsignificant findings. Future investigations should account for opioid exposure using multivariate analyses to enhance the accuracy of pain-outcome assessments.

Compared to prior research, our findings are consistent with published literature suggesting that minimally invasive SIJ fusion improves pain with minimal complications [18,19]. One large center study comparing open SIJ fusion with minimally invasive techniques found shorter operative times and length of stay, as well as less blood loss, as a benefit of minimally invasive SIJ fusion [18]. This same study by Smith et al. found that one and four-year pain scores improved by around 3 points higher in the minimally invasive surgical group when controlled for age, sex, and other parameters [18]. A smaller study found similar results when comparing open and minimally invasive SIJ fusion, though the University of Minnesota’s SIJ population experienced longer operative times and greater blood loss with the open technique, ultimately suggesting that minimally invasive approaches lead to better pain outcomes [19]. Other clinical benefits of a minimally invasive approach include improved quality of life, decreased blood loss, and reduced surgical time [6].

Our study contributes to this body of evidence by specifically evaluating robotic assistance, a newer innovation designed to enhance trajectory consistency regarding screw placement, mitigate soft tissue damage, and minimize radiation exposure [20]. Despite these advantages, the robotic approach also introduces considerations related to cost, operative efficiency, and workflow integration. While early cases may involve longer setup times, refinement in technique and team familiarity can mitigate this burden.

Limitations

This study was a retrospective, single-center analysis, which may introduce selection bias. An important limitation of this study is the imbalance in preoperative opioid use between the NA and RA groups, likely confounding our pain outcome comparisons. Additionally, two patients were lost to follow-up, and their clinical follow-up data were excluded per exclusion criteria. Another limitation is the lack of uniform long-term follow-up among patients, which restricts our ability to assess the durability of outcomes beyond the early postoperative period. Additionally, the potential influence of the surgeon's learning curve on RA SIJ fusion must be acknowledged. Early cases may have been associated with longer operative times and higher technical variability, which could have impacted the outcomes reported here. As experience with robotic techniques increases, these variables may improve, and future studies should account for this factor when comparing surgical modalities. The small sample size may limit the power to detect statistically significant differences in postoperative outcomes, and future multi-center, prospective studies with larger sample sizes and extended follow-up durations are warranted to validate these findings and explore the differences in long-term outcomes in greater detail.

## Conclusions

The NA and RA groups exhibited no significant differences in postoperative pain scores or 30-day complication rates despite lower postoperative pain scores in the RA group, demonstrating the feasibility of the RA technique, with safety and patient outcomes comparable to traditional minimally invasive techniques. The longer operative time observed in the RA group may be associated with the early adoption of this technology at our institution. Given the small sample size, retrospective design, and potential confounding factors related to preoperative opioid use, these findings should be considered as preliminary. Larger prospective multicenter studies with longer follow-up are needed to confirm the safety, efficacy, and long-term benefits of RA SIJ fusion.
